# Atlanta Society of Medicine

**Published:** 1891-04

**Authors:** 


### ATLANTA SOCIETY OF MEDICINE.

ATLANTA, GA., March 3, 1891.

Society called to order, President F. W. McRae in the chair.

The president announced that the subject for discussion for the evening was Pneumonia, and asked Dr. Duncan to open the discussion.

Dr. Duncan said the plan he follows in the early stages of pneumonia, is to give tinct. veratrum in one to four drop doses, according to pulse, and some form of opium with quinine, as Dover's powder grains three to five quinine grains three every three or four hours. If bowels are open he does not give cathartic, as patients with pneumonia do not bear it well. Says he does not blister in the early stage, but later, if the case is not progressing favorably, sometimes blisters. He uses, in some cases, turpentine and lard to the chest, also poultices, and seems to get good results from them. He rarely sees cases in which blood-letting is called for, but thinks in cases where there is great engorgement bleeding would be beneficial, but that patients usually pass this stage before seen by physicians.

After acute stage has passed, he gives some form of iodine, usually potas. iodide, in decided doses, or syrup hydriodic, acid, in drachm doses. In the stage of convalescence, gives one of the preparations of hypophosphites, as Fellows'. He gives liberal diet, which he says is better borne than in typhoid fever. If called for, gives alcoholics.

Dr. Richardson said he used veratrum in the first five or six years of his practice with fair results, and while it is true that we can, with that drug, reduce the pulse from 120 to 70 or 80, the respiration from 40 to 24, and the temperature from 103 or 104 degrees to 100 degrees, yet he believes with Flint, that pneumonia is a constitutional and self-limited disease, and the patient is in no better condition after taking the drug than before. Says that veratrum is used more in the treatment of pneumonia in the South than in the North. He is opposed to blistering in the early stage of the disease. He thinks quinine the best antipyretic unless the temperature goes above 103

degrees, when phenacetin may be given. Says the most important thing is to keep the patient quiet with opium, and the temperature of the room at about 60 degrees F.

Dr. Parks says he begins by giving calomel and opium, but does not give veratrum, and never blisters in the early stage. Later in the course of the disease gives ammon. carb. and potas. iod.

Dr. Kendrick thinks the main indications are to reduce the temperature and sustain the heart action. Says old people die from failure of right heart, and the three most important remedies are opium, quinine and alcohol.

Dr. Johnson says there is a question whether or not pneumonia is a constitutional and self-limited disease, and the treatment is modified thereby. He does not consider it self-limited or constitutional, but a local inflammatory disease, and says if he blisters at all, it is in the early stage. He uses a mixture of ammonia, turpentine and lard to posterior part of chest, and, in adults, gives veratrum, but in children prefers to use tinct. aconite in one-fourth or one-half drop doses every few minutes until temperature is reduced. He gives opium and quinine, and has given phenacetin. Gives simple diet and alcoholics if called for.

Dr. Howell said his experience in the treatment of pneumonia was quite limited, but would rely upon veratrum, guarded by opium, quinine and blistering in the early stage, if at all.

Dr. Kennedy says he gives opium in the first stage; later alcohol is the main dependence. Says the chest should be well protected, and thinks poultices and oiled silk the best means of protection.

Dr. Hollis says he follows the plan indicated by Dr. Richardson. He thinks the disease self-limited.

Dr. Hood says he has treated many cases, and his plan is to swath the chest with cotton batting and cover with oiled silk. He then gives ammon. muriat. and camphor water, and, unless the temperature goes above 103 degrees, pays no attention to it. If it goes high, gives salicylates. Later in disease, gives alcoholics if necessary.

Dr. Hardon says he does not treat pneumonia, but while in general practice it was his rule to reduce the temperature by sponging the patient from head to foot with cold water. Later gave stimulants. He thinks the disease self-limited.

Dr. Childs says he has treated few cases in private practice, but while house surgeon to city hospital, Charleston, the main dependence was upon ammon. carb., tinct. digitalis and alcohol, with poultice to chest.

Dr. McRae says he considers pneumonia an essentially specific and self-limited disease. He does not think the temperature of much consequence, and has seen no good in reducing it. In fact, it has been his experience that cases do best in which the temperature runs high. Says he believes with Dr. A. H. Smith, that the danger in pneumonia is from failure in and engorgement of the right heart, and that in sthenic cases, where there is great engorgement, bleeding is indicated. He uses opium only in the early stage, and does not think digitalis indicated, as it causes contraction of the capillaries, and heightens arterial tension, the very thing we want to avoid. He withholds liquids as much as it is consistent with comfort of patient. He has never used blisters, and is afraid of veratrum, as it has a weakening influence on the heart. Thinks nux vomica the best tonic in these cases.

Dr. Duncan says he has aborted pneumonia with veratrum after it has reached the stage of brick-dust expectoration and crepitant rale. In his own case, he was out on the fourth day. He thinks it perfectly safe if watched; says it acts through the pneumogastric nerve, and in his hand seems to possess specific properties in this disease. Has used capsicum with quinine and comp. Dover powder with good results. Has blistered in early stage, but will never do so again.

---

SPECIAL MEETING, MARCH 12, 1891.

This meeting was called that the profession of Atlanta, and especially the members of the Society, might have the opportunity of meeting Dr. W. C. Baily, of New York, and hear his views on the diagnosis and treatment of tuberculosis by Koch's new remedy, "tuberculin."

The president explained the object of the meeting, and introduced Dr. Bailey, who he said was, at the request of many

Southern physicians, visiting Southern cities explaining and demonstrating the new remedy for tuberculosis, Koch's lymph.

Dr. Bailey thanked the Society and the medical fraternity of Atlanta for the hearty response to the invitation to meet him ; said he had hoped to be able to show the application of the lypmph, but notwithstanding the kindness of physicians in trying to get a subject for him, he had failed in obtaining one suitable for the experimeut.

Said it was assumed that we of the profession had familiarized ourselves with the literature on the subject, and that it was also recognized that little was *known* on the subject, and the discoverer himself is content to submit it to the test of time—the *only true test* of all new remedies. Said we all have the greatest confidence in the integrity and ability of the great discoverer, and that our duty is not to criticise but to assist Dr. Koch in his scientific researches. His object, he said, was not to discuss results, but the best methods to adopt in using a very dangerous and as yet only an experimental therapeutic agent.

Perateoloid, he said, was the name given the new remedy by Dr. Koch, but that some one else had given it the name of "tuberculin," by which name it is now known. Dr. Bailey showed the apparatus, as made by Koch, for applying the remedy, which, he said, like the inventor, was very simple though effective.

Said the lymph was very susceptible to the influence of germs, therefore it was necessary to sterilize the fingers, syringe, etc., and for this purpose alcohol is used.

Said the lymph is a red solution, which comes in small vials, of which you make a 10 per cent. solution of distilled water, and 1-2 per cent. carbolic acid. Then make a 1 per cent. solution of this 10 per cent. solution, of which the dose is 1 mm., or less than one drop.

The point of inoculation selected by Koch is the back, simply because it is less painful to the patient. It is not necessary to inoculate at point of disease.

Dr. Bailey says the selection of cases is a very important matter. If the temperature is above 100 deg. F., it is not safe to use the remedy, as the reaction would be too intense, and if the patient is very greatly emaciated it is not safe, because the patient will not react from the effect of the treatment. Says it is not contra-indicated in general tuberculosis, but if the lungs are greatly involved, the reaction will be too intense, and the amount of necrotic tissue will be too great to be gotten rid of. The treatment should not be tried in patients suffering from diarrhœa.

Says in New York, at the hospital with which he is connected, they have reports of fifty-six cases treated, in none of which have there been any deaths or unfavorable symptoms, and in many there has been decided improvement, while six have been discharged apparently cured. Says in one case of lupus of nose of 23 years standing, in which the cartilages were involved, there was complete cure. In two cases laryngeal phthisis, in which the voice was entirely lost, the voice was restored and patients cured.

Says that one benefit to the profession from the use of "tuberculin," is the establishment of the law that the larynx is almost never involved alone, and that in cases of lung tuberculosis the larynx is often involved, and that in skin and joint cases the larynx is often found involved when it was not suspected before. Says it is fully demonstrated that the reaction only takes place in tubercular tissue; therefore it is a valuable diagnostic.

Says febrile reaction is not essential to the beneficial results of tuberculin; in fact, cases in which the febrile reaction is least, with greatest amount of local change, give best results. Said in one case of lupus of larynx the temperature fell below normal after inoculation, and this was one of the cases in which got best results.

Says the local reaction is more rapid and more marked in some cases than in others. Says that in lupus erythematous the reaction is less, slower than in lupus vulgaris, and in the former has never seen a case of cure, although there is improvement.

In lupus vulgaris the local changes are increased redness, pain and swelling, and a copious serous exudation. The exudation forms crusts or scabs, which alterwards fall away, the tubercular tissue dies and sloughs, and comes away, leaving below healthy granulations. In one case of tuberculosis of wrist and hand, except index finger, there was so much pain the first night after inoculation that the patient did not sleep more than two hours. The next day the pain ceased, and the exudation dripped from the whole hand, except the index finger, which was not involved. Can only be used in the early stage of lung tuberculosis for reasons already given. Dr. Bailey says it is too soon to form our judgment on the matter, but acquaintance with the remedy stimulates hope.

Dr. Hardon asked how often the injections are made, and Dr. Bailey answered, every forty-eight hours to a week. Said never give more than 10 mm. at once, and give just often enough to get reaction without causing rise of temperature. Said if the temperature goes above 100, to wait until it falls, if it is a week. Said each case must be treated and studied alone, for no formulated rule can be laid down to apply to all cases. Said patients with tuberculosis of lungs can take larger doses than when the pleura only is involved, and that it could not be used at all in tubercular meningitis.

Dr. Kendrick asked if failure to get reaction is positive proof that tuberculosis is not present. Dr. Bailey said no, for the remedy might fail to reach the disease ; but that getting the reaction was positive proof of the presence of tubercular tissue. Said in one case of cancer repeated inoculations had failed to get any reaction whatever, and in case of supposed cancer in young medical student the remedy was tried, and the reaction was well marked, proving that the disease was not cancer, but tuberculosis. In this case there was a cure.

Dr. Stockton asked if it had been tried in Potts' disease. Dr. Bailey said yes, with intense reaction. Dr. Stockton also asked if it had been tried in syphilis. Dr. Bailey said it had, "but that it does not touch syphillis."

Dr. Gaston wished to know if any benefit had been obtained in the treatment of scrofula with tuberculin. Dr. Bailey said it had been tried with good results in some cases.

